# Quality-of-life outcomes before and after multilevel pharyngeal surgery in moderate-to-severe obstructive sleep apnea

**DOI:** 10.1016/j.bjorl.2026.101866

**Published:** 2026-07-22

**Authors:** Na Hua, Yujing Fan, Yanzhe Lyu, Guili Deng, Jizhe Wang, Xiuli Liu

**Affiliations:** a Dalian Medical University, Department of Otorhinolaryngology, Dalian, China; b Affiliated Zhongshan Hospital of Dalian University, Department of Otorhinolaryngology, Dalian, China

**Keywords:** Obstructive sleep apnea, Quality of life, Vertigo, Sleep medicine

## Abstract

**Objectives:**

To assess changes in subjective quality of life (dizziness, tinnitus, sleep quality, daytime sleepiness) in moderate-to-severe obstructive sleep apnea patients before and after multilevel pharyngeal surgery (uvulopalatopharyngoplasty, bilateral tonsillectomy and tongue-base radiofrequency ablation), and identify the main factors influencing postoperative outcomes.

**Methods:**

Thirty-one OSA patients diagnosed via Polysomnography (PSG) underwent preoperative bilateral bithermal caloric testing and completed the Dizziness Handicap Inventory (DHI), Tinnitus Handicap Inventory (THI), Pittsburgh Sleep Quality Index (PSQI), and Epworth Sleepiness Scale (ESS). Assessments were repeated 6-months postoperatively. Paired*t*-tests compared pre/post scores, and multiple linear regression identified factors affecting quality of life.

**Results:**

Our analysis revealed three major findings: (1) Canal Paresis (CP) was not correlated with DHI scores; (2) Postoperative DHI, THI, PSQI, and ESS scores significantly decreased (p ≤ 0.05). The 95% confidence intervals for the differences were 2.396–8.830, 0.773–3.356, 2.835–4.907, and 3.376–7.140, respectively; (3) Apnea-Hypopnea Index (AHI) and Lowest Oxygen Saturation (LSpO_2_) were primary determinants of postoperative quality of life. AHI and LSpO_2_ strongly negatively correlated with postoperative DHI (βAHI = −0.604, βLSpO_2_ = −0.485) and PSQI scores (βLSpO_2_ = −0.656, βAHI = −0.384).

**Conclusions:**

The subjective quality of life in moderate to severe OSA patients, particularly for dizziness, tinnitus, sleep quality, and daytime sleepiness, was significantly improved after surgical treatment. In terms of postoperative quality of life, AHI and LSpO_2_ were identified as the main factors. Higher preoperative AHI and lower LSpO_2_ were associated with better postoperative outcomes.

**Level of evidence:**

2.

## Highlights

• Quality-of-life gains after multilevel surgery in moderate-to-severe OSA.
• DHI, THI, PSQI and ESS all dropped significantly postoperatively.
• AHI and LSaO_2_ were the main factors influencing postoperative quality of life.
• Higher baseline AHI plus lower LSaO_2_ yielded better outcomes.

## Introduction

Obstructive Sleep Apnea(OSA) is a prevalent sleep-disordered disease characterized by recurrent upper airway obstruction during sleep, accompanied by snoring, apnea, sleep deprivation, and chronic intermittent hypoxia, leading to multi-system damage.^1^ Previous studies^2^ have shown that chronic intermittent hypoxia in OSA can severely affect the brainstem and vestibular system, leading to vestibular dysfunction such as dizziness, vertigo, and instability, which in turn can cause falls and related fractures,^3^ significantly impacting daily life. The current study employed a pre-post self-controlled design, involving 31 patients with moderate to severe OSA. We assessed the changes in quality of life before and after surgical treatment using questionnaires, including the Dizziness Handicap Inventory (DHI), Tinnitus Handicap Inventory (THI), Pittsburgh Sleep Quality Index (PSQI), and Epworth Sleepiness Scale (ESS). Additionally, we explored the main factors influencing postoperative quality-of-life improvement. Currently, there is a paucity of studies on the impact of OSA on quality of life.^4^

## Methods

### Patients and study design

A total of 31 patients with moderate to severe OSA, diagnosed by Polysomnography (PSG), were enrolled in this study. All patients underwent multilevel pharyngeal surgery, including uvulopalatopharyngoplasty, bilateral tonsillectomy, and tongue-base radiofrequency ablation, at the Department of Otorhinolaryngology, Affiliated Zhongshan Hospital of Dalian University, between January 2023 and December 2023. Follow-up continued until June 2024. This self-controlled design aimed to assess the impact of multilevel surgery on quality of life by comparing each patient's preoperative and postoperative status. The study was conducted in accordance with the Declaration of Helsinki (as revised in 2013) and was approved by the Institutional Review Board of Chinese Clinical Trial Registry (registration number ChiCTR2500102351). Written informed consent was obtained from all participants at the time of enrollment. Data were collected at baseline and at the 6-month follow-up. The preoperative assessment included PSG reports and bilateral bithermal caloric test results. Both preoperatively and at 6-months postoperatively, patients completed the DHI, THI, PSQI, and ESS.

Inclusion criteria included patients aged 18–65 years, with moderate-to-severe OSA defined by Apnea Hypopnea Index (AHI) of ≥15 events/h as shown by PSG monitoring, who were scheduled to undergo multilevel pharyngeal surgery and were able to understand and complete questionnaires.

Exclusion criteria included: peripheral or central vertigo; severe cardiovascular, cerebrovascular, endocrine, or psychiatric disorders; a history of head trauma, ear surgery, or otologic diseases; significant occupational noise exposure; and use of any medications known to impair the cochleo-vestibular system or to affect sleep.

### Polysomnography (PSG)

All the participants underwent a full ‒ night PSG study with at least 7-hs of recording time (Alice 6 Diagnostic Sleep System; Philips Healthcare, the Netherlands). Sleep staging was scored according to the criteria of the American Academy of Sleep Medicine (AASM) published in 2012.^6^ Apnea was defined as the cessation of airflow for ≥ 10 seconds. Hypopnea was defined as a ≥30% reduction of airflow lasting ≥10 seconds and associated with a ≥4% decrease in oxyhemoglobin saturation. The Apnea-Hypopnea Index (AHI) was derived from the total number of apneas and hypopneas per hour of sleep. Additional parameters recorded included the Longest Apnea Duration (LAD), maximum heart rate, and Lowest Oxygen Saturation (LSpO_2_).

### Bilateral bithermal caloric test

A standard caloric test was performed on each subject using the vestibular function testing system (Vestibular Examination Unit, Interacoustics A/S, Denmark). Cold (24 °C) and hot (50 °C) air was used for irrigation, with cold air applied first. The maximum slow-phase velocity of nystagmus was recorded for 10 seconds after each irrigation, and Canal Paresis (CP) and Directional Preponderance (DP) were calculated. CP ≥ 25% and DP ≥ 30% were considered abnormal.

### Questionnaires

In this study, international standard questionnaires and scoring systems of DHI, THI, PSQI, and ESS were used for assessment before and 6-months after surgery.

### Dizziness Handicap Inventory (DHI)

Developed by Jacobson and Newman, the DHI consists of 25-items to assess the quality of life of dizziness patients in physical, functional, and emotional aspects. The validated Chinese version of the questionnaire was used in this study.^7^

### Tinnitus Handicap Inventory (THI)

Developed by Newman, the THI consists of 25-items and three dimensions (functional, emotional, and catastrophic) to understand the impact of tinnitus on patients' personal lives and emotions. A THI score higher than zero indicates the presence of tinnitus symptoms, with higher scores indicating more severe tinnitus.^8^

### Pittsburgh Sleep Quality Index (PSQI)

The PSQI consists of 18-self-assessment items to evaluate the subjective sleep quality and sleepiness of patients in the past month.^9^ Higher scores indicate poorer sleep quality.

### Epworth Sleepiness Scale (ESS)

Developed by Johns, the ESS consists of 8 questions to assess the likelihood of dozing off in different situations, mainly to evaluate the degree of sleepiness.^10^ The total score is 24, and a score >9 indicates the presence of sleepiness.

### Surgical protocol

**Fig. 1:**
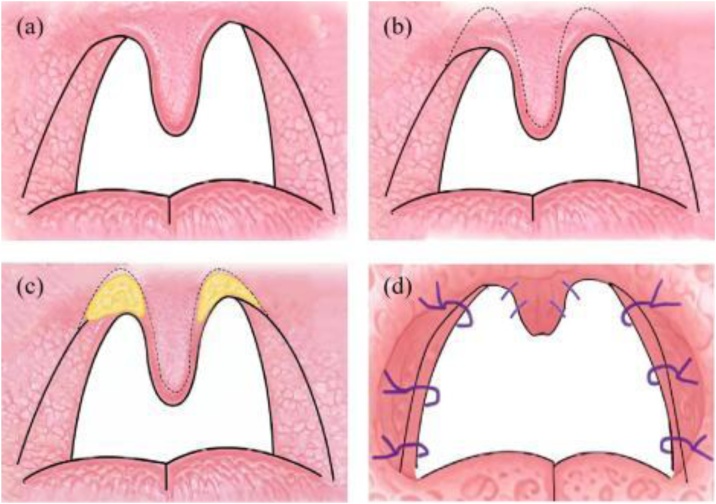
Han’s Uvula-Preserving UPPP technique. (a) Intraoperative view of the oropharynx, demonstrating the uvula and soft palate. (b) Partial resection of the soft palate and lateral pharyngeal wall mucosa with preservation of the uvula. (c) Wound closure showing the enlarged anteroposterior and transverse diameters of the pharyngeal airway. (d) Immediate postoperative view of the oropharynx.

**Fig. 2:**
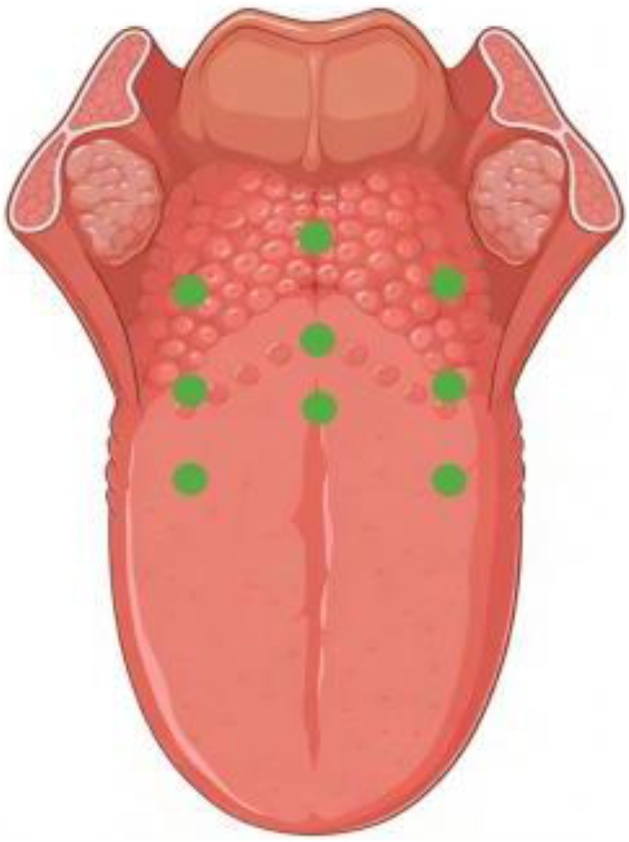
3 × 3 radio-frequency ablation pattern at the base of tongue.

All procedures were performed under general anaesthesia with orotracheal intubation and a Boyle–Davis mouth gag. Pre-operatively, the modified Mallampati classification, Friedman stage and Brodsky tonsil grade (III–IV in every case) were documented to confirm multilevel surgical candidacy. Every patient then underwent an identical, single-session multilevel operation by the same surgeon comprising: (1) Bilateral low-temperature plasma tonsillectomy with the AC401 handpiece; (2) Uvula-preserving uvulopalatopharyngoplasty (Han’s technique) (Fig. 1)^5^; and (3) Tongue-base radiofrequency ablation using a disposable handpiece (AC304), with energy delivered in a 3 × 3 grid pattern across nine points: three midline sites (1 cm anterior, 2 cm anterior, and 1 cm posterior to the foramen caecum) and three corresponding points on each lateral third of the tongue base (Fig. 2). No concomitant nasal surgery was required, as the nasal cavities were patent.

### Statistical analysis

All statistical analyses were performed using SPSS for Windows, version 25.0 (IBM, Armonk, NY, USA). The descriptive statistics of the indicators for patients with moderate to severe OSA were expressed as $\bar{\text{X}}$ ± S. The changes in DHI, THI, PSQI, and ESS scores before and after surgery were analyzed using paired *t*-tests. The correlation between preoperative DHI, THI, PSQI, ESS scores and bilateral bithermal caloric test CP and DP values was analyzed using Pearson or Spearman correlation tests. The impact of preoperative indicators on postoperative questionnaire scores was analyzed using multiple linear stepwise (backward) regression analysis.

## Results

### Basic information and PSG results of patients with moderate to severe OSA

Among the 31 patients with moderate to severe OSA, there were 21 males and 10 females. The basic information and PSG results are as follows in Table 1.

**Table 1:** Basic information and PSG results of patients with moderate to severe OSA.

Variable	$\bar{\text{X}}\pm \text{S}$	Range
Age (years)	40.87 ± 8.36	28‒60
Duration of illness (months)	107.03 ± 85.34	6‒240
AHI (events/h)	56.82 ± 27.45	15.9‒101.6
LSpO_2_ (%)	73.77 ± 13.93	43‒89
LAD (s)	71.81 ± 76.13	14.0‒459.5
Maximum heart rate (beats/min)	183.81 ± 50.38	91‒255
BMI	28.56 ± 3.41	20.9‒35.1

PSG, Polysomnography; OSA, Obstructive Sleep Apnea; AHI, Apnea-Hypopnea Index; LSpO_2_, Lowest Oxygen Saturation; LAD, Longest Apnea Duration; BMI, Body Mass Index.

### Preoperative bilateral bithermal caloric test results and DHI, THI, PSQI, ESS scores and analysis of the correlation between them

Abnormal CP values were observed in 12 patients (38.71%, 12/31), and abnormal DP values were observed in 7 patients (22.6%, 7/31).

The questionnaire results showed that among the 31 patients with moderate to severe OSA, 9 had normal DHI scores, 20 had mild impairment, 2 had moderate impairment, and 0 had severe impairment, with 22 patients (70.97%, 22/31) having abnormal scores. For THI scores, 16 patients had normal scores, 13 had mild symptoms, 1 had moderate symptoms, 0 had severe symptoms, and 1 had catastrophic symptoms, with 15 patients (48.39%, 15/31) having abnormal scores. For PSQI scores, 7 patients had scores ≤5, and 24 patients had scores >5. For ESS scores, 12 patients had mild sleepiness, 5 had moderate sleepiness, and 14 had severe sleepiness.

The correlation analysis between questionnaire scores and bilateral bithermal caloric test CP and DP values showed that preoperative THI scores were positively correlated with DHI scores (*r* = 0.430, p = 0.016), and preoperative PSQI scores were positively correlated with ESS scores (*r* = 0.492, p = 0.005). There was no correlation between CP and DP values in the bilateral bithermal caloric test and the scores of DHI, THI, PSQI, and ESS.

### Comparison of DHI, THI, PSQI, and ESS scores before and after surgery in patients with moderate to severe OSA

After multilevel surgery, the scores of DHI, THI, PSQI, and ESS were significantly reduced compared with those before surgery, suggesting a possible improvement in subjective perception, with the differences being statistically significant in Table 2.

**Table 2:** Changes in questionnaire scores before and after surgery in patients with moderate to severe OSA (Mean ± SD, Points).

Group	DHI	THI	PSQI	ESS
Preoperative	9.81 ± 13.85	7.61 ± 16.60	8.10 ± 3.32	12.61 ± 6.81
Postoperative	4.19 ± 6.54	5.55 ± 13.92	4.23 ± 2.01	7.36 ± 4.66
*t*	−3.643	−3.087	7.628	5.705
p	0.000	0.002	0.000	0.000
95% CI for difference	2.396∼8.830	0.773∼3.356	2.835∼4.907	3.376∼7.140

DHI, Dizziness Handicap Inventory; THI, Tinnitus Handicap Inventory; PSQI, Pittsburgh Sleep Quality Index; ESS, Epworth Sleepiness Scale.

### Multivariate analysis of the impact of preoperative indicators on postoperative subjective perception in patients with moderate to severe OSA

The postoperative questionnaire scores of patients with moderate to severe OSA were analyzed using multiple linear stepwise (backward) regression analysis with preoperative indicators (age, BMI, duration of illness, CP, DP, preoperative maximum heart rate, LSpO_2_, AHI). The results showed that the factors affecting postoperative DHI scores included AHI, LSpO_2_, and preoperative maximum heart rate, with AHI and LSpO_2_ being highly correlated with postoperative DHI scores (β_AHI_ = −0.604, β_LSpO2_ = −0.485), showing a negative correlation. The factors affecting postoperative PSQI scores included LSpO_2_, AHI, and CP, with LSpO_2_ and AHI being highly correlated with postoperative PSQI scores (β_LSpO2_ = −0.656, β_AHI_ = −0.384), showing a negative correlation. The factor affecting postoperative ESS scores was the LAD, which was positively correlated. It can be seen that AHI and LSpO_2_ were the main factors affecting postoperative subjective perception in Table 3.

**Table 3:** Regression coefficient estimates and test results of multiple linear regression equations.

Item	β	Beta	*t*	p
DHI Scale				
AHI	−0.144	−0.604	−2.680	0.012
LSpO_2_	−0.228	−0.485	−2.144	0.041
Preoperative maximum heart rate	0.048	0.369	2.232	0.034
PSQI Scale				
LSpO_2_	−0.095	−0.656	−3.102	0.004
AHI	−0.028	−0.384	−1.782	0.086
CP	0.027	0.323	2.048	0.050
ESS Scale				
LAD	0.037	0.598	4.021	0.000

CP, Canal Paresis.

### Postoperative safety and complications

Among the 31 patients who underwent multilevel pharyngeal surgery, 11 (35.5%) experienced mild throat pain or dysphagia within 1–2 weeks; all symptoms were self-limiting and required no intervention. No occurrences of bleeding, infection, velopharyngeal insufficiency, airway compromise, or cardiovascular events were observed during the follow-up period.

## Discussion

We performed bilateral bithermal caloric tests on 31 patients with moderate to severe OSA and found that 38.71% (12/31) of patients had abnormal canal paresis values. The DHI questionnaire results showed that 70.97% (22/31) of patients experienced dizziness, vertigo, or unsteadiness to varying degrees in their daily activities. Simple correlation analysis showed no significant correlation between CP and DHI, indicating that most patients with moderate to severe OSA experienced subjective vestibular symptoms, but only some had objective vestibular function abnormalities. This suggests that there are some uncertain factors affecting the subjective vestibular perception of patients with moderate to severe OSA during daily activities. Previous studies have found that although vestibular dysfunction is more prevalent in OSA patients, nocturnal hypoxia may not lead to otolith dysfunction in OSA patients.^11^ In recent years, studies have reported a close relationship between sleepiness, nighttime sleep apnea, and dizziness.^12^ Sowerby et al. compared 46 patients with chronic idiopathic dizziness, 20 patients with benign paroxysmal positional vertigo, and 69 patients with hearing loss but no dizziness, and found that the chronic idiopathic dizziness group had significantly higher scores for sleepiness and nighttime sleep quality tests than the other groups.^13^ This suggests that sleep disorders affect the perception of dizziness. The ESS is a reliable and effective scale for assessing sleepiness, and the PSQI is a commonly used scale for evaluating sleep quality. ESS and PSQI are often used to assess the sleep status of OSA patients. In this study, the ESS and PSQI were used to survey 31 patients with moderate to severe OSA, and the results showed that the preoperative average ESS score was 12.61 ± 6.81 and the average PSQI score was 8.10 ± 3.32, indicating that patients with moderate to severe OSA had significant sleep disorders. Current research shows that OSA is the most common sleep breathing disorder, with a high incidence rate in the population. Its fragmented sleep and sleep deprivation lead to sleepiness, memory, attention, and motor coordination impairments, affecting patients' balance during daily activities.^14^ Therefore, sleep disorders and dizziness often coexist and form a vicious cycle in patients with moderate to severe OSA, severely affecting the quality of life.

Multilevel surgery is an important method to relieve upper airway obstruction in patients with moderate to severe OSA. Our study found that 6-months after multilevel surgery, the scores of DHI, PSQI, ESS, and THI were significantly reduced, indicating that multilevel surgery effectively improved patients' dizziness, tinnitus, nighttime sleep quality, and sleepiness, and improved the quality of life. The role of multilevel surgery in improving AHI and LSpO_2_ in patients with moderate to severe OSA has been widely recognized. Previous studies have reported that hypoxia not only affects the cardiovascular, nervous, and endocrine systems but also has certain effects on the cochlear, vestibular, and brainstem.^15^ Animal evidence indicates the vestibular nerve nucleus is particularly hypoxia-sensitive.^16^ Furthermore, sleep deprivation can affect the vestibular system and induce vestibular dysfunction, with even brief periods impairing the vestibular nucleus and spatial perception.^12^ Even brief sleep deprivation can cause vestibular nerve nucleus dysfunction and affect spatial position perception. Therefore, after multilevel surgery, the improvement of LSpO_2_ and the reduction of apnea and hypopnea can significantly improve patients' dizziness, tinnitus, sleep quality, and sleepiness. However, the ideal state of improvement in the long term still needs further research.

To further explore the factors affecting the degree of improvement in quality of life such as dizziness and sleep after surgery, this study included patients' age, BMI, duration of illness, CP, maximum heart rate, LAD, LSpO_2_, and AHI in multiple linear stepwise (backward) regression analysis. The results showed that the factors affecting postoperative dizziness efficacy included AHI, LSpO_2_, and maximum heart rate, the factors affecting postoperative sleep quality efficacy included LSpO_2_, AHI, and CP, and the factor affecting postoperative sleepiness efficacy was the LAD. It can be seen that AHI and LSpO_2_ were the main factors affecting the quality of life of patients with moderate to severe OSA after surgery. Concurrently, we found that AHI and LSpO_2_ were negatively correlated with the improvement of postoperative dizziness and sleep quality, and the LAD was positively correlated with the improvement of postoperative sleepiness. That is, the higher the preoperative AHI and the higher the preoperative LSpO_2_, the more obvious the improvement in postoperative dizziness and sleep disorder subjective perception. Previous studies have shown that sleep apnea is a comorbidity that exacerbates vestibular dysfunction. Participants with sleep apnea have more severe vestibular symptoms than those without sleep apnea.^17^ Another study reported that postural stability depends on the coordination between the central nervous system and visual, proprioceptive, and vestibular information.^18^ Sleep deprivation has been proven to affect this function. Sleep deprivation in OSA patients affects ear nerve function, further leading to postural control disorders. The above studies show that the more severe the AHI, the more obvious the subjective feeling of dizziness and instability. After multilevel surgery, AHI is significantly reduced, sleep deprivation is reduced, and the subjective feeling of patients is significantly improved. Regarding the impact of nocturnal hypoxia on the vestibular function of OSA patients, Ulusoy B et al. studied the relationship between vestibular evoked myogenic potentials and the severity of OSA, and found that the VEMP wave elicitation rate in the moderate and severe OSA groups was significantly lower than that in the mild OSA group, indicating that the more severe the hypoxia, the greater the impact on the brainstem and vestibular system.^2^ Another study reported that more severe hypoxemia is associated with better subjective sleep quality in OSA patients, suggesting that hypoxemia affects related perceptual disorders, which is not significant in mild hypoxemia but becomes significant in severe hypoxemia.^19^ It can be seen that severe hypoxemia in moderate to severe OSA has a great impact on the vestibular system and perceptual system of the body. Although the hypoxic state is improved after multilevel surgery, the vestibular and sleep disorder perceptual systems still need more time to recover. Therefore, in patients with moderate-to-severe OSA who are intolerant or unwilling to use CPAP, multilevel surgery should be considered without undue delay to correct AHI and LSpO_2_, improve symptoms such as dizziness, sleep quality, and sleepiness, and prevent patients from experiencing falls, traffic accidents, and other related events.

Our study had several limitations. First, it should be noted that all patients included in this analysis had moderate to severe OSA. Thus, our findings may not apply to patients with mild OSA. Second, this study lacks postoperative Polysomnography (PSG) data to objectively confirm the physiological improvement in OSA severity following surgery. Third, the study results were evaluated using subjective questionnaires, with a lack of additional objective indicators. Finally, the sample size was small, and some confounding factors that may affect postoperative efficacy were not fully assessed in the multiple linear regression analysis. We will continue to investigate these factors in our future work.

## Conclusions

In conclusion, our study demonstrates that effective intervention to improve AHI and LSpO_2_ ‒ the main factors influencing postoperative quality of life ‒ is associated with significant gains in subjective well-being for patients with moderate-to-severe OSA. For those who are intolerant or unwilling to use CPAP, timely consideration of effective alternative therapies, such as multilevel surgery, can be valuable in mitigating disease burden and enhancing quality of life.

## ORCID ID

Na Hua: 0009-0003-7032-0448

Yujing Fan: 0009-0007-0416-0609

Yanzhe Lyu: 0009-0005-1903-6630

Guili Deng: 0009-0001-2217-3303

Xiuli Liu: 0000-0002-2629-0603

## Financial support

The authors declare that this study received no financial support.

## Data availability statement

The datasets used and/or analysed during the current study are available from the corresponding author on reasonable request.

## Declaration of competing interest

The authors declare no conflicts of interest.
